# Frequency and economic burden of psychogenic non-epileptic seizures in patients applying for disability benefits due to epilepsy

**DOI:** 10.1055/s-0042-1759759

**Published:** 2022-12-28

**Authors:** Mehmet Taylan Peköz, Kezban Aslan-Kara, Turgay Demir, Gulfem Aktan, Mehmet Balal, Soner Cakmak, Hacer Bozdemir

**Affiliations:** 1 Çukurova University, Faculty of Medicine, Department of Neurology, Epilepsy Unit, Adana, Türkiye.; 2 Çukurova University, Faculty of Medicine, Department of Neurology, Adana, Türkiye.

**Keywords:** Psychogenic Nonepileptic Seizures, Epilepsy, Retirement, Electroencephalography, Financial Stress, Convulsões Psicogênicas não Epilépticas, Epilepsia, Aposentadoria, Eletroencefalografia, Estresse Financeiro

## Abstract

**Background** Psychogenic non-epileptic seizures (PNES) resemble epileptic seizures and are often misdiagnosed as epilepsy.

**Objective** To investigate the frequency of PNES and to calculate the economic burden of the patients who admitted to video-electroencephalographicmonitoring (VEM) to obtain a diagnosis of epilepsy in order to apply for disability retirement.

**Methods** The present retrospective study included 134 patients who required disability reports between 2013 and 2019 and had their definite diagnoses after VEM. Following VEM, the patients were divided into three groups: epilepsy, PNES, and epilepsy + PNES.

**Results** In total, 22.4% (n = 30) of the patients were diagnosed with PNES, 21.6% (n = 29) with PNES and epilepsy, and 56% (n = 75), with epilepsy. The frequency of PNES among all patients was of 44% (n = 59). In patients with PNES alone, the annual cost of using anti-seizure medication was of 160.67 ± 94.04 dollars; for psychostimulant drugs, it was of 148.3 ± 72.48 dollars a year; and the mean direct cost for diagnostic procedures was of 582.9 ± 330.0 (range: 103.52–1601.3) dollars.

**Conclusions** Although it is challenging to determine the qualitative and quantitative total cost in these patient groups, early diagnosis and sociopsychological support will reduce the additional financial burden on the health system and increase the quality of life of the patients.

## Resumo

**Antecedentes** As crises psicogênicas não epilépticas (CPNE) se assemelham a crises epilépticas, e muitas vezes são diagnosticadas erroneamente como epilepsia.

**Objetivo** Investigar a frequência de CPNE e calcular o impacto econômico dos pacientes internados para serem submetidos a monitoramento videoencefalográfico (MVE) para obter um diagnóstico de epilepsia e requerer aposentadoria por invalidez.

**Métodos** Este estudo retrospectivo incluiu 134 pacientes que solicitaram laudo médico de incapacidade entre 2013 e 2019, e obtiveram seus diagnósticos definitivos após serem submetidos a MEV. Os pacientes foram divididos em três grupos: epilepsia, CPNE, e epilepsia + CPNE.

**Resultados** Após o MEV, 22,4% (n = 30) dos pacientes foram diagnosticados com CPNE, 21,6% (n = 29), com CPNE + epilepsia, e 56%, com epilepsia. A frequência de CPNE entre todos os pacientes foi de 44% (n = 59). Em pacientes somente com CPNE, o custo anual do uso de anticonvulsivantes foi de US$ 160,67 ± 94,04; para os psicoestimulantes, o custo anual foi de US$ 148,3 ± 72,48; e a média do custo direto de procedimentos diagnósticos foi de US$ 582,9 ± 330,0 (variação: 103,52–1601,3).

**Conclusões** Embora seja um desafio determinar o custo total qualitativo e quantitativo nesses grupos de pacientes, o diagnóstico precoce e o apoio sociopsicológico reduzirão o impacto financeiro adicional ao sistema de saúde e aumentarão a qualidade de vida dos pacientes.

## INTRODUCTION

Epilepsy is a chronic disorder that may result in working disability. 1 Psychogenic non-epileptic seizures (PNES) resemble epileptic seizures; however, they lack neurobiological origin, and are not associated with the electrophysiological alterations observed in epilepsy. 1 2 Video-electroencephalographic Monitoring (VEM) is the gold-standard method to differentiate these two conditions. 3

The estimated annual incidence of PNES was reported as 1.4 to 4.9/100 thousand people, and its estimated prevalence in the general population is of 2–33/100 thousand. 4 5 A total of 5% to 10% of the patients admitted to the outpatient clinics of epilepsy centers and 20% to 40% of the patients monitored have PNES. 4 6

Epilepsy is related to high direct and indirect costs; the indirect costs are due to unemployment and early disability. 7 The patients diagnosed with PNES are faced with a high level of unemployment and low income. 8 9 On the other hand, establishing the diagnosis of PNES may take long, 4 10 and the patients may not be able to work or find a job until the diagnosis has been made, which results in high indirect costs, similar to those faced by patients with epilepsy. 8

The patients with PNES take anti-seizure medication (ASM) and/or psychostimulant drugs (PSSDs) unnecessarily due to misdiagnosis and inappropriate treatment of this condition, and they are exposed to adverse effects of the redundant medications. In addition to the adverse effects, the medications also result in high financial costs for this group of patients. There is limited information in the literature about the economic burden of PNES. 11 12 13

The present study aims to analyze the frequency of PNES as well as the direct and indirect costs of the patients who admitted to VEM for obtain a diagnosis of epilepsy in order to apply for disability retirement.

## METHODS

The present single-center retrospective cohort study was carried out in a tertiary healthcare center in Adana, southern Türkiye, after obtaining approval from the l Ethics Committee for Non-invasive Clinical Research of the Faculty of Medicine of Çukurova University (decision no: 2021/109).

The study included 134 adult patients referred to the Epilepsy Unit of the Department of Neurology of Çukurova University between January 2013 and December 2019 who undergo VEM to confirm the diagnosis of epilepsy, determine the frequency of seizures in patients with a history of epilepsy, and to assess their disability.

The patients were most frequently referred from other hospitals and from the Turkish Social Security Institution (SSI). Our center is the reference institution in our region authorized by the Ministry of Health to issue medical board reports. The patients were referred to confirm the diagnosis of “epilepsy” and to be hospitalized for a minimum of 2 weeks, following the criteria of the Ministry of Family and Social Policies on “Regulation on Disability Assessment for Adults” (no. 20.02.2019/30692). 14 The SSI requests information about the follow-up of VEM patients with VEM within the aforementioned period, including recordings of their seizures, disclosure of the ictal and postictal findings, and an assessment of the time it takes for the patients to return to their daily work in the postictal period.

### Patient selection and VEM

The subjects who met all of the following criteria were included:

- Age ≥ 17 years;
- Having an SSI request for diagnosis and frequency of seizures after VEM;
- Having undergone VEM for five days or more;
- History of seizure determined at the outpatient clinic;
- Admission for the diagnosis of epilepsy;
- Patients wishing to apply for disability retirement due to the diagnosis of epilepsy;
- History of more than four monthly episodes of seizure; and
- Patients assessed by a psychiatrist.

All patients were monitored with a 64-channel electroencephalography (EEG) system (EEG 1200, Nihon Kohden, Shinjuku-ku, Tokyo, Japan). Scalp electrodes were placed according to the standard international 10–20 system and with the guidelines of the American Clinical Neurophysiology Society. 15

The patients presented to the outpatient clinic with the request for a medical report and had their histories taken; then, they were hospitalized and followed-up in VEM Unit of the Neurology Clinic with the prediagnoses of epilepsy, PNES, or epilepsy + PNES. The seizures defined by the patient and their relatives were recorded in the VEM Unit, the patient records (hospitalization period in the requested report: two to four weeks) were video-monitored [VM]) in another room, and the follow-up was completed.

After the follow-up with VEM and VM, we categorized the patients' into three groups according to their diagnoses: epilepsy, epilepsy + PNES, and PNES. Patients were included in the epilepsy group when ictal EEG and associated seizure patterns were observed on VEM; subjects were included in the PNES group when associated with a typical non-epileptic episode without ictal EEG; and the epilepsy + PNES group was composed of patients in whom an epileptic seizure was observed with a non-epileptic episode. However, in addition to non-epileptic episodes, patients who did not have an ictal period during the follow-up but had abnormal EEG, radiological, or pathological/abnormal neurological examination findings and a history of ictal period were semiologically included in the epilepsy + PNES group.

The clinical findings and demographic features of all patients were extracted from their medical files and discharge summaries. Age at onset, family history of epilepsy, risk factors for epilepsy, frequency of episodes, history of status epilepticus, kind and duration of the use of ASMs or psychiatric drugs were recorded for every patient, as well as the result of the neurological examination (normal or abnormal) and the electrophysiological and brain magnetic resonance imaging (MRI) findings (normal, abnormal). The duration of the video-EEG recording, the time of occurrence of the epileptic seizures or PNES (day, night), the number of seizures or events and their duration were also recorded. The costs of the medicines were calculated as the dollar equivalent of the lowest label price of the drugs in Turkish lira determined by the SSI.

All patients were examined throughout their hospitalization periods by two senior epileptologists, and the VEM records were manually reviewed by two senior epileptologists. The psychiatric assessment was performed by a trained psychiatrist who was blinded to the epilepsy diagnosis during hospitalization. All the patients were assessed using axes I and II of the Structured Clinical Interview for the Diagnostic and Statistical Manual of Mental Disorders, Fifth Edition (SCID).

According to the study design, 34 patients were excluded because they had been hospitalized for fewer than 4 days.

### Statistical analysis

The statistical analyses were performed using the Statistical Package for the Social Sciences (IBM SPSS Statistics for Windows, IBM Corp., Armonk, NY, US) software, version 20.0. The categorical variables were expressed as frequencies and percentages, and the continuous variables, as mean and standard deviation values. The Chi-squared test was used to compare the categorical variables, and the comparison of means was performed with the Student *t* -test or the Mann-Whitney U test, where appropriate. Statistical significance was set as *p* < 0.05 for all tests.

## RESULTS

A total of 168 patient files were reviewed. According to the study design, 34 patients were excluded because they had been hospitalized for fewer than 4 days. Of the 134 patients, 119 were male (88.8%), with a mean age of 39.3 ± 11.3 (range: 17–65) years. All of the patients had been referred from another center for a diagnosis of epilepsy. After the history was obtained in the outpatient clinic, the patients were admitted to the VEM Unit with the prediagnoses of epilepsy (59.7%), PNES (3.7%) and PNES + epilepsy (36.6%). The mean length of hospital stay was of 11.9 ± 4.2 (range: 5–30) days. Following VEM and VM, 56% (n = 75) of the patients were diagnosed with epilepsy, 22.4% (n = 30), with PNES, and 21.6% (n = 29), with PNES + epilepsy, and their disability reports were prepared. Almost all of those patients, except for 3 (2.2%), were on antiepileptic treatment, the mean duration of medication usage was of 18.3 ± 12.9 years, and the mean monthly frequency of seizures was of 5.8 ± 5.5 according to the first history obtained. The demographic characteristics of the patients are presented in Table 1 .

**Table 1:** Demographic and clinical characteristics of the study sample

		Epilepsy: n (%)	PNES: n (%)	PNES + epilepsy: n (%)	Total: n (%)	*p*
Patients: n (%)		75 (56%)	30 (22.4%)	29 (21.6%)	**134**	
Gender: male/female		66/9	27/3	26/3	119 (88.8)/15 (11.2)	0.94
Age in years (mean ± SD)		39.7 ± 11.2	35.5 ± 11.8	42.2 ± 10.2	39.31 ± 11.3 (17-65)	0.06
Age at seizure onset (mean ± SD)		18.7 ± 12.1	24.4 ± 14.7	23.5 ± 14.8	21.1 ± 13.5	**0.03**
Risk factors for epilepsy	Febrile convulsion	18 (54.5)	9 (27.3)	6 (18.2)	33 (24.6)	0.6
	Head trauma	29 (51.8)	17 (30.4)	10 (17.9)	56 (41.8)	0.2
	Difficult delivery	3 (4)	2 (6.7)	3 (10.3)	8 (6)	0.4
	CNS infection	2 (2.7)	0	2 (6.9)	4 (3)	0.3
	CVD	4 (5.3)	0	5 (55.6)	9 (6.7)	**0.02**
	Space-occupying lesion (benign/malignant)	7 (9.3)	2 (6.7)	0	9 (6.7)	0.2
	Family history of epilepsy (%)	14.7	13.3	13.8		0.9
Prediagnosis in outpatient clinic: n (%)	Epilepsy	64 (80)	9 (11.3)	7 (8.8)	80 (59.7)	**< 0.001**
	PNES	1 (20)	4 (80)	0	5 (3.7)	
	PNES + epilepsy	10 (20.4)	17 (34.7)	22 (44.9)	49 (36.6)	
ASMs taken: n (%)	None		3 (10)		3 (2.3)	**< 0.001**
	1	24 (32)	17 (56.7)	11 (37.9)	52 (38.8)	
	≥2	51 (68)	10 (33.3)	18 (62.1)	79 (58.9)	
Monthly frequency of seizures: mean ± SD	Before monitoring	5.01 ± 4.8	5.1 ± 7.2	6.9 ± 6.5	5.5 ± 5.8	0.3
	During monitoring					
Seizure frequency/month mean ± SD (min.–max.)		1.83 ± 2.8 (0–15)		0.9 ± 1.6 (0–7)	1.2 ± 2.3 (0–15)	**0.001**
PNES frequency/month mean ± SD (min.–max.)			1.4 ± 1.9 (0–8)	3.3 ± 6.1 (0–30)	1.04 ± 3.2 (0–30)	
Time of event: n (%)	Daytime	35 (46.7)	21 (70)	16 (55.1)	72 (53.7)	**0.01**
	Night	0 (0)	2 (6.7)	2 (6.9)	4 (2.9)	
	Asleep + awake	40 (53.3)	7 (23.3)	11 (37.9)	58 (43.3)	
Years of ASM use (mean ± SD)		20.1 ± 11.7	8.7 ± 9.6	19.1 ± 11.9	17.5 ± 12.2	**< 0.001**
Abnormal neurological examination: n (%)		22 (29.3)	5 (13.3)	8 (27.6)	35 (26.1)	0.18
Abnormal EEG: n (%)		63 (84)	11 (36.7)	24 (75.9)	94 (73.1)	**< 0.001**
Abnormal MRI: n (%)		59 (78.7)	5 (16.7)	18 (58.6)	82 (61.2)	**< 0.001**

Abbreviations: ASM, anti-seizure medication; CNS, central nervous system; CVD, cerebrovascular disease; EEG, electroencephalography; MRI, magnetic resonance imaging; PNES, psychogenic non-epileptic seizure; SD, standard deviation;.

Note: Values of *p* ≤ 0.05 were considered statistically significant and marked in bold in the table.

Although there was a predominance of male patients in all three groups, neither gender distribution ( *p* = 0.94), mean age ( *p* = 0.2), age at seizure onset ( *p* = 0.08), family history of epilepsy ( *p* = 0.9), monthly frequency of seizures ( *p* = 0.3), nor the findings of the neurological examination ( *p* = 0.18) were statistically different among them. However, the duration of ASM usage was longer in the epilepsy and epilepsy + PNES groups ( *p* < 0.001), and abnormal EEG and MRI findings were also significantly different in these two groups ( *p* < 0.001 for both).

The seizures were recorded during the day or had a diurnal pattern in the epilepsy group. In the PNES and epilepsy + PNES groups, since some attacks were recorded after VEM was completed and only when VM recording was available, the attacks that occurred in the evening were recorded as nightly or attacks, since the wake pattern could not be determined. Thus, it was also determined that the episodes in patients with PNES could occur at night. Seizures were frequently asleep + awake in patients with epilepsy and were also frequently recorded during the day in patients with PNES ( *p* = 0.01).

A total of 18.6% (n = 25) of the patients were using antidepressants, and 11.2% (n = 15), antipsychotic drugs. The use of PSSDs was significantly higher in the PNES group compared to the other two groups ( *p* = 0.05).

The SCID I and II showed that 15.9% of the patients had conversion disorder, 4.5%, psychosis, 4.5%, mental retardation (3 in group 1, and 1 in group 3), 3.4%, depressive disorder, 2.3%, epileptic personality disorder, 1.1%, antisocial personality disorder (in group 3), 1.1%, obsessive personality disorder, and 1.1%, panic disorder.

We found that the longest duration of ASM use in the PNES group was of 38 (mean: 8.7 ± 9.65) years ( *p* **<** 0.001); however the duration ASM use was longer among the epilepsy and epilepsy + PNES groups ( *p* = 0.05) ( Table 3 ), and the cost of drug usage was statistically significantly higher in the epilepsy group ( *p* **<** 0.001; Table 3 ). Long-term PSSD use was evident in all t groups ( *p* = 0.390). We observed that PSSD was started approximately 10 years after the diagnosis of epilepsy; however, in PNES patients, they were started at the onset of symptoms or around that time. In PNES patients, the mean annual cost of ASM use was of 160.67 ± 94.04 dollars, and the mean cost of PSSD use was of 148.3 ± 72.48 dollars. The mean cost of all diagnostic procedures and follow-up during the hospital stay was of 582.9 ± 330.0 (range: 103.52–1601.3) dollars. The cost of the hospital stay comprised laboratory examinations (complete blood count, serum drug levels, routine blood biochemistry), VEM, radiological imaging, consultations, hospitalization, and patient care parameters.

**Table 2:** Psychiatric comorbidities and type of psychostimulant drug used among the study sample

		Epilepsy (n = 75; 56%)	PNES (n = 30; 22.4%)	PNES + epilepsy (n = 29; 21.6%)	*p*
Psychiatric comorbidity	No: n (%)	60 (80)	16 (53.3)	19 (65.5)	**0.04**
	Yes: n (%)	15 (20)	14 (46.7)	10 (34.5)	
Psychostimulant drug	Antidepressants: n (%)	7 (9.3)	10 (33.3)	8 (27.5)	**0. 05**
	Antipsychotics: n (%)	9 (12)	4 (13.3)	2 (6.9)	0.7

Note: Value of *p* ≤ 0.05 were considered statistically significant and marked in bold in the table.

**Table 3:** Annual cost in US dollars and years of drug use per diagnosis

	Epilepsy	PNES	PNES + epilepsy	*p*
Years of ASM use: mean ± SD (min.–max.)	20.16 ± 11.75 (1–58)	8.7 ± 9.65 (1–38)	19.14 ± 11.9 (1–51)	**< 0.001**
Years of PSSD use: mean ± SD (min.–max.)	10.16 ± 8.65 (1–30)	6.25 ± 6.13 (1–20)	7.37 ± 5.28 (1–15)	0.390
Annual cost of ASM use: mean ± SD (min.–max.)	224.33 ± 112.47 (19.44–618.48)	160.67 ± 94.04 (24.24–423.6)	198.99 ± 110.92 (24.24–495.96)	**0.027**
Annual cost of PSSD use: mean ± SD (min.–max.)	352.66 ± 380.59 (27.84–965.28)	148.3 ± 72.48 (57.96–274.8)	92.88 ± 23.77 (77.76–129.6)	0.096

Abbreviations: ASM, anti-seizure medication; PSSD, psychostimulant drug; SD, standard deviation.

Note: Value of *p* ≤ 0.05 were considered statistically significant and marked in bold in the table.

## DISCUSSION

The present study, based on diagnoses confirmed through VEM and psychiatric evaluations, showed the prevalence of PNES (22.4%), epilepsy (56%), and epilepsy + PNES (21.6%); the overall frequency of PNES was of 44%.

Studies 4 6 have reported that 5% to 10% of the outpatients in epilepsy clinics and 20% to 40% of inpatients in epilepsy monitoring units have PNES. Yon et al. 6 stated that 10.2% out of 1,983 patients who underwent VEM had PNES; moreover, 44.8% of these patients had definite PNES, 32.5% had definite PNES + epilepsy, and the remaining patients had disorders of other subgroups. 6 Benbadis et al. 16 reported that, during the follow-up of patients diagnosed with PNES, 9.4% were found to have concomitant epilepsy. In the present study, we found a higher prevalence (44%) of PNES and epilepsy + PNES and previous studies 4 reported a ratio ranging from 10% to 20%. In the present study and in the one by Yon et al., 6 the patients had similar sociocultural characteristics, and both studies found that the frequency of PNES was higher in patients followed up with VEM; we suppose that the rate of PNES was higher in the present study because the patients who requested disability reports were included, and they had ulterior motives. However, Asadi-Pooya et al., 17 in a multicenter, international, and cross-cultural study, reported that PNES patients share more similarities than differences.

As shown in the present study, a significant difference was found between the frequency of seizures reported by the patient and their relatives and the frequency of seizures observed during the clinical follow-up. Although there was no significant difference in the epilepsy group, the frequency of seizures reported in the history was higher in the epilepsy + PNES and PNES groups than in the clinical follow-ups. We evaluated that this may be explained by the ulterior motives of the patients when requesting the disability reports.

According to the historical features, 55% of all the patients were defined generalized seizures. It is noteworthy that 83.3% of the patients in the PNES group were defined as generalized seizures according to the semiological features reported by the patients or their relatives. Most of the attacks in patients with PNES were indicated as generalized seizures.

Although it is hard to distinguish between PNES and epileptic seizures, it has been estimated that the delay in the diagnosis of PNES can range from 3 to 8.4 years. 4 10 18 19 Delay in diagnosis causes unnecessary use of antiepileptics, resulting in both adverse effects and a socioeconomic burden. 17 In the present study, PNES patients started to use PSSDs in the year of symptom onset[, but epilepsy patients started using PSSDs long after the diagnosis of epilepsy ( Table 3 ). It seems that patients with epilepsy needed psychiatric support due to the clinical conditions created by the unresolved disease, stigmatization, poor quality of life, unemployment, and low income.

Patients with PNES are common in epilepsy centers, filling almost 40% of VEM Units and costing an estimated 650 million dollars annually. 8 Studies conducted in Ireland 12 and in the United States 20 have shown that the annual cost for PNES patients is similar to that of chronic epilepsy patients until they are diagnosed. However, both studies are from high-income countries, and there are no data on the economic burden of PNES in developing countries such as Türkiye. For PNES, the estimated yearly total cost of direct medical expenses per person was calculated as €5,429.30 in Ireland 12 and as US$8,156 in the United States. 11 In the present study, we calculated an average cost of US$582.9 ± 330.0 (range: 103.52–1,601.3) per person for direct medical expenses during the period of hospitalization and follow-up for the disability assessment. On the other hand, we determined the annual cost per person as US$308.97 due to the use of ASM + PSSD in the PNES patients. Unlike the aforementioned Irish study, 12 the present study was not based on pre-VEM health expenses, and we only calculated the cost of hospitalization for diagnosis and the cost of use of ASM +PSSD before the diagnosis was made. Therefore, we determined that if a person is hospitalized once a year, there is an annual cost of US$891.87 (minimum amount: US$582.9 + US$308.97) for their diagnosis and the medical treatment.

In 1994, Begley et al. 21 calculated the cost per patient after the diagnosis of epilepsy as US$4,272 for patients in remission and as US$138,602 for those with resistant epilepsy; an approximate cost was calculated, and, in a study published 21 years later, in 2015, Begley and Durgin 22 emphasized that these costs were mostly related to the use of ASMs. In a study conducted in China, 13 the authors emphasized that the annual direct cost per patient was of US$372, the costs due to loss of productivity were of US$289, and a large part of the direct cost was also due to the use of ASMs. In the present study, since the patients themselves requested disability reports with the diagnosis of resistant epilepsy, it would be inevitable that the annual costs per PNES patient would be as high as the costs for those with resistant epilepsy had the correct diagnosis not been made.

In the present study, compared to epileptic patients, PNES patients had more psychiatric comorbidities ( *p* = 0.04; Table 2 ), which included conversion disorder, psychosis, depressive disorder, personality disorders, panic disorder, and intellectual disability. Our results are in agreement with those of the literature; PNES patients have a higher risk of developing posttraumatic stress disorder, personality disorder, and anxiety, but not depression. 23 Scévola et al. 23 showed that 100% of PNES patients had psychiatric comorbidities, and the analysis of 32 studies by Diprose et al. 2 revealed that this rate ranged from 53% to 100% in PNES patient. In the present study, the rate of psychiatric comorbidities was higher in PNES patients (46.7%) than in the epilepsy group.

In the present study, we observed that PNES patients started using PSSDs earlier than those with epilepsy and almost at the same time they started using ASMs ( Table 3 ). Consistent with the findings of the study conducted by Hantke et al., 24 we determined that PNES patients frequently used benzodiazepines and antipsychotic drugs and were followed up with at least two or more drug combinations. For ASM, in the study by Zanzmera et al., 25 28.1% of the PNES patients were using ASMs, whereas, in the present study, only 2.2% of patients were not using ASMs.

In previous studies, 26 27 there was a predominance of the female gender among PNES patients, but, in the study, most of the patients were male. The fact that active workers in Turkish society are predominantly male and that men have to have a regular income to provide for their household was believed to be the reason for the high number of requests for disability reports by male patients.

We found that the comorbid PNES rate was high in chronic epileptic patients. This may be related to the ulterior motive of these patients, who want a disability report to get disability retirement. That is why the VEM in such patients is significant to confirm the diagnosis of epilepsy and comorbid conditions.

In conclusion, all patients included in the present study were diagnosed with epilepsy before their admission to our VEM Unit, and most of them were diagnosed with PNES or comorbid PNES after monitoring. Physicians should definitely consider PNES in patients with resistant epilepsy and comorbid psychiatric diseases. Therefore, it would be suitable to refer the patients who are not clinically in remission and who meet the criteria for the use of PSSDs to tertiary centers for VEM as soon as possible. Thus, unnecessary use of medications, associated high costs, and adverse effects may be prevented.

In addition, long-term follow-up and psychosocial support to patients diagnosed with PNES will provide crucial information concerning their prognosis, their tendency to quit or continue using ASMs, as well as support for employment and the possibility for resolving the disease.

### Limitation of the study

Due to the retrospective nature of the present study, the expenses incurred in the last year before VEM could not be determined. Since the active substance of the drugs used by the patients was recorded in their medical files, the cost of that drug was calculated based on the preparation with the lowest market value. However, we know that preparations containing the same active substance may have different prices in our country. Therefore, we supposed that the annual cost for each patient in terms of drugs could be higher.
